# Supplementary material for: The Efficacy of Additives for the Mitigation of Aflatoxins in Animal Feed: A Systematic Review and Network Meta-Analysis
Source: Toxins (Basel). 2022 Oct 15;14(10):707. doi: 10.3390/toxins14100707 (PMC9607122; doi:10.3390/toxins14100707)
Supplement: Supplementary file 1 [file toxins-14-00707-s001.zip › toxins-1960091-supplementary.pdf]

Supplementary Materials: The efficacy of additives for the mitigation of aflatoxins in animal feed: a systematic review and network meta-analysis

Oluwatobi Kolawole, Wipada Siri-Anusornsak, Awanwee Petchkongkaw, Julie Meneely and Christopher Elliott

Table S1. Database search strategy with keywords to retrieve eligible articles for systematic review and network meta-analysis.

Database	Search keywords	Number of articles
Scopus	DOCTYPE (ar) TITLE (poultry* OR chicken* OR broiler*) ABS (clay* OR "clay mineral" OR modified* OR "modified clay" OR agriculture* OR "agricultural waste" OR fibres* OR "fibers" OR "yeast" OR polymers* OR plants* OR enzyme* OR bacteria* OR fungi* OR "yeast cell wall" OR glucomannan* OR "antioxidant") ABS (feed*) ABS (aflatoxin*)	228
Web of Science	(((TI=(poultry* OR chicken* OR broiler*)) AND AB=(clay* OR “clay mineral" OR modified* OR "modified clay" OR agriculture* OR "agricultural waste" OR fibres* OR “fibers” OR "yeast" OR polymers* OR plants* OR enzyme* OR bacteria* OR fungi* OR "yeast cell-wall" OR glucomannan* OR antioxidant*)) AND AB=(feed*)) AND AB=(aflatoxin*)	221
PubMed	((((poultry*[Title] OR chicken*[Title] OR broiler*[Title])) AND (clay*[Title/Abstract] OR “clay mineral" [Title/Abstract] OR modified* [Title/Abstract] OR "modified clay" [Title/Abstract] OR agriculture* [Title/Abstract])) OR "agricultural waste" [Title/Abstract] OR fibres* [Title/Abstract] OR “fibers” [Title/Abstract] OR "yeast" [Title/Abstract] OR “yeast cell wall” [Title/Abstract] OR polymers* [Title/Abstract] OR plant* [Title/Abstract] OR enzyme* [Title/Abstract] OR bacteria* [Title/Abstract] OR fungi* [Title/Abstract] OR "yeast cell wall" [Title/Abstract] OR glucomannan* [Title/Abstract] OR antioxidant* [Title/Abstract] AND (feed*[Title/Abstract])) AND (aflatoxin*[Title/Abstract])	561

Table S2. The network estimates for all possible treatment comparisons with effect size estimates and confidence intervals

V1	V2	V3	V4
Antioxidant	-1.69 (-3.63; 0.25)	2.70 (-9.94; 15.34)	-0.54 (-2.93; 1.84)
-1.82 (-3.45; -0.18)	Control	2.99 (0.00; 5.98)	1.60 (-0.55; 3.75)
1.01 (-1.77; 3.80)	2.83 (0.38; 5.28)	Inorganic Binder	-0.81 (-4.96; 3.35)
-0.29 (-2.09; 1.51)	1.53 (-0.14; 3.19)	-1.30 (-3.92; 1.32)	Organic Binder

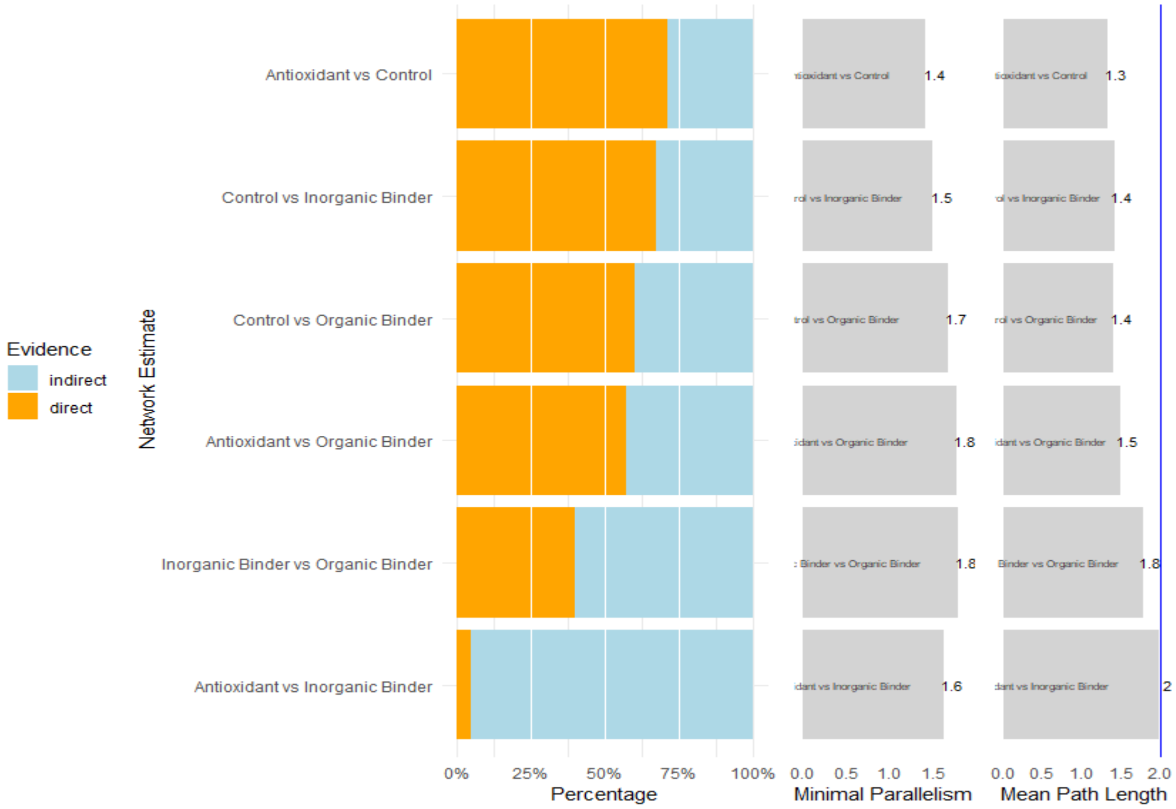

Figure S1. Plot showing the percentage of direct and indirect evidence used for each estimated comparison.

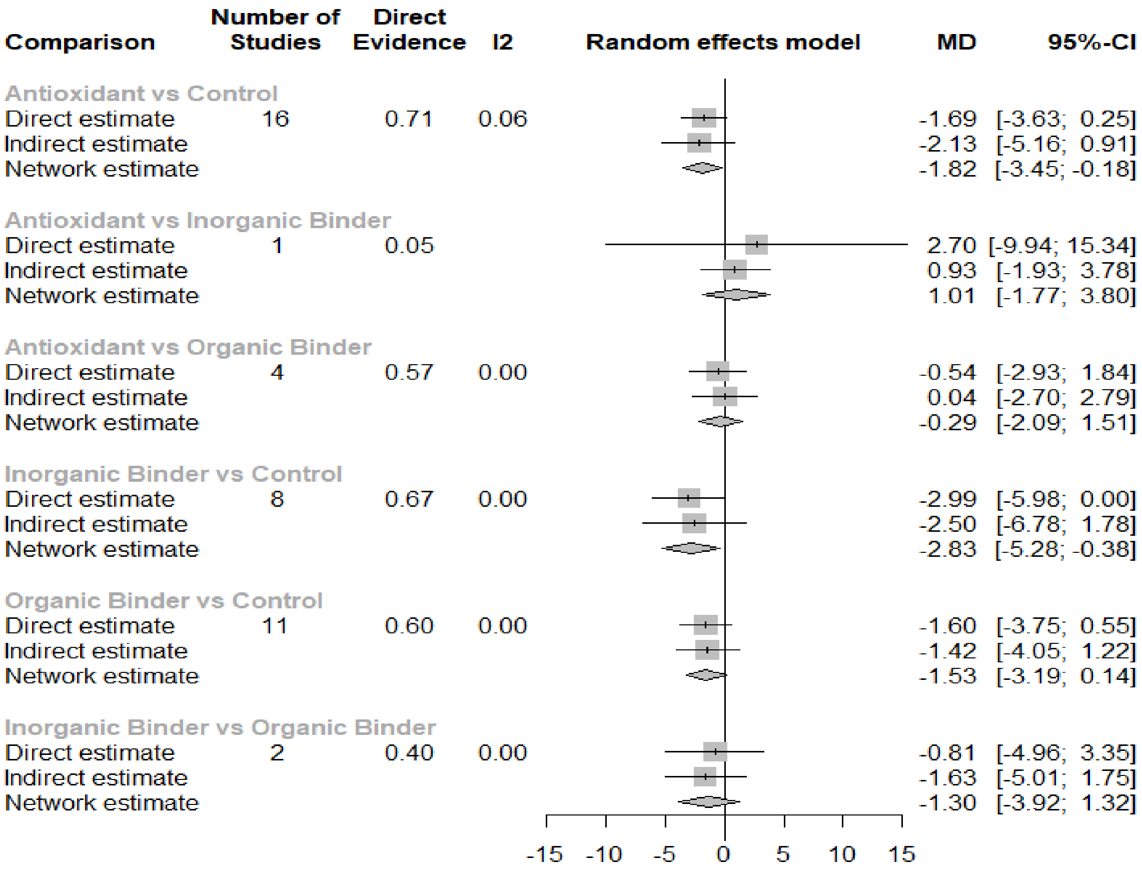

Figure S2. Netsplit forest plot showing the consistency of contribution of direct and indirect evidence estimates of individual comparisons in our network

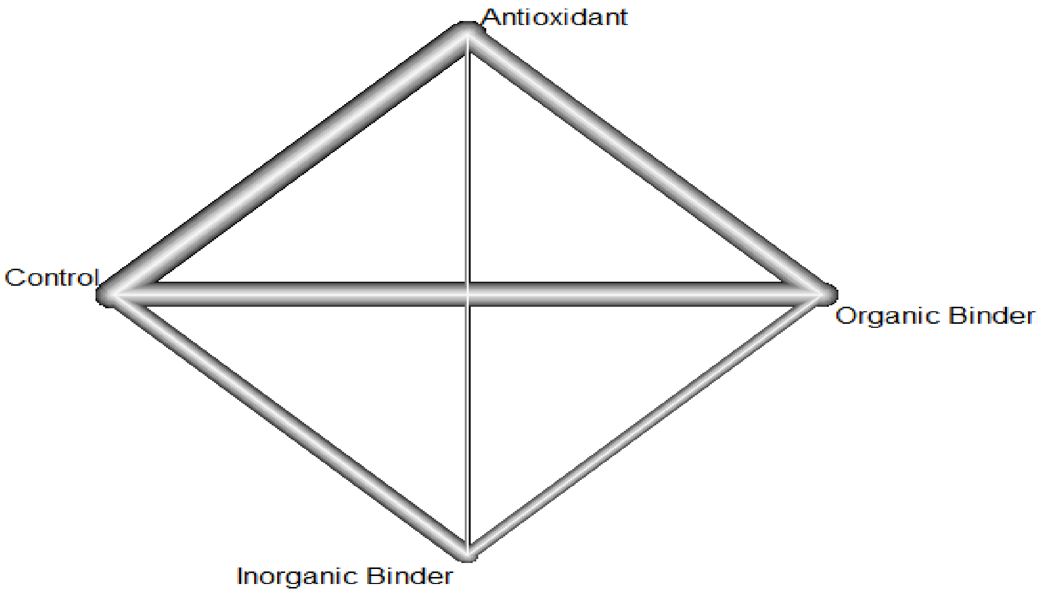

Figure S3. Network graph of treatment comparisons. The thicknesses of the lines correspond to the number of comparisons.
